# Co-located quantitative trait loci mediate resistance to *Agrobacterium tumefaciens, Phytophthora cinnamomi*, and *P. pini* in *Juglans microcarpa* × *J. regia* hybrids

**DOI:** 10.1038/s41438-021-00546-7

**Published:** 2021-05-01

**Authors:** Ramesh K. Ramasamy, Ming-Cheng Luo, Charles A. Leslie, Dianne Velasco, Natalia Ott, Ali McClean, Abhaya M. Dandekar, Mallikarjuna Aradhya, Patrick J. Brown, Gregory T. Browne, Daniel A. Kluepfel, Andreas Westphal, Jan Dvorak

**Affiliations:** 1 Department of Plant Sciences, University of California, Davis, USA; 2 USDA-ARS Crops Pathology and Genetics Research Unit, Department of Plant Pathology, University of California, Davis, USA; 3 National Clonal Germplasm Repository, USDA-ARS, University of California, Davis, USA; 4 Department of Nematology, University of California, Riverside, USA

**Keywords:** Plant hybridization, Plant breeding, Plant hybridization

## Abstract

Soil-borne plant pathogens represent a serious threat that undermines commercial walnut (*Juglans regia*) production worldwide. Crown gall, caused by *Agrobacterium tumefaciens*, and Phytophthora root and crown rots, caused by various *Phytophthora* spp., are among the most devastating walnut soil-borne diseases. A recognized strategy to combat soil-borne diseases is adoption of resistant rootstocks. Here, resistance to *A. tumefaciens, P. cinnamomi*, and *P. pini* is mapped in the genome of *Juglans microcarpa*, a North American wild relative of cultivated walnut. Half-sib *J. microcarpa* mother trees DJUG 31.01 and DJUG 31.09 were crossed with *J. regia* cv. Serr, producing 353 and 400 hybrids, respectively. Clonally propagated hybrids were genotyped by sequencing to construct genetic maps for the two populations and challenged with the three pathogens. Resistance to each of the three pathogens was mapped as a major QTL on the long arm of *J. microcarpa* chromosome 4D and was associated with the same haplotype, designated as haplotype *b*, raising the possibility that the two mother trees were heterozygous for a single Mendelian gene conferring resistance to all three pathogens. The deployment of this haplotype in rootstock breeding will facilitate breeding of a walnut rootstock resistant to both crown gall and Phytophthora root and crown rots.

## Introduction

Persian (English) walnut (*Juglans regia*) is an important nut tree crop worldwide. China, Iran, and the United States lead in world production of commercial walnuts (FAOSTAT), while California produces virtually all of the US crop. Commercial walnut orchards often incur serious losses from soil-borne pathogens, primarily *Agrobacterium tumefaciens* (causal agent of crown gall), numerous *Phytophthora* spp. (Phytophthora root and crown rots), *Armillaria mellea* (Armillaria root rot), and phytopathogenic nematode species (root lesion, root-knot, and ring nematodes).

The strategy of cultivating scion varieties on disease-resistant rootstocks, usually interspecific hybrids or wild relatives, has been effective in managing soil-borne diseases. About 80% of commercially grown walnuts in California are grafted onto Paradox rootstocks, which are hybrids derived from the locally adapted Northern California black walnut, *J. hindsii*, pollinated with *J. regia* pollen^1^. Additional, commercially available, hybrid rootstocks include ‘RX1’ (a clonally propagated *J. microcarpa* × *J. regia* hybrid), selected for resistance to *Phytophthora* spp.^2^, and ‘VX211’ (a *J. hindsii × J. regia* hybrid), tolerant to lesion nematode (*Pratylenchus vulnus*).

Screening a collection of hybrids of diverse species of *Juglans* with *J. regia* revealed resistance to both *A. tumefaciens* and *Phytophthora* spp. in *J. microcarpa*^2,3^, a small statured tree native to dry regions of Texas, New Mexico, Oklahoma, and Kansas. *Juglans microcarpa* and *J. regia* diverged about 8 million years ago^4^, but they readily hybridize. The hybrids are fully male sterile and ~90–99% female sterile. Both species have *n* = 16 chromosomes, which originated by a whole-genome duplication of an ancestral genome with *n* = 8 chromosomes^5^.

Resistance to *A. tumefaciens* has been observed in a number of woody perennials, such as *Vitis* spp.^6,7^, *Prunus* spp.^8^, *Malus* spp.^9,10^, and *Rosa* spp.^11^, in addition to *Juglans* spp.^3^, as well as in herbaceous plants, such as *Pisum sativum*^12^ and *Arabidopsis thaliana*^13^. In *A. thaliana*, resistance to *A. tumefaciens* was shown to be due to a deficiency in T-DNA integration^13,14^. Resistance to *Phytophthora cinnamomi* in woody perennials was investigated in Japanese and Chinese chestnuts (*Castanea crenata* and *C. mollissima*, respectively), where it was mediated by two major quantitative trait loci (QTLs) and several minor QTLs^15,16^. The molecular mechanism of this resistance has not been determined.

QTL mapping relies on the detection of association between a phenotypic trait and genetic markers^17^. Single nucleotide polymorphisms (SNPs) are currently nearly universally used as genetic markers. SNPs have been discovered and characterized in the *J. regia* genome^18–20^. Due to the divergence of the *J. microcarpa* and *J. regia* genomes, *J. regia* SNP markers are not useful for mapping the *J. microcarpa* genome. The genotyping-by-sequencing (GBS) approach^21^ is a viable strategy for developing new SNP markers for genotyping *J. microcarpa* × *J. regia* hybrids and the construction of maps of both genomes.

This paper describes the development of two populations of *Juglans* hybrids created by crossing *J. microcarpa* half-sib mother trees DJUG 31.01 (henceforth 31.01) and DJUG 31.09 (henceforth 31.09) with the cultivated *J. regia* cv. Serr. These populations were genotyped by GBS, and genetic maps of the parental genomes were constructed. Segregation for resistance to *A. tumefaciens, P. cinnamomi*, and *P. pini* among the hybrids was analyzed in each mapping population (MP), and resistance was mapped as QTLs. An assembled and annotated *J. microcarpa* genome sequence^4^ was used to analyze the QTLs.

## Results

### GBS marker development and linkage map construction

The F_1_ hybrids 31.01 × cv. Serr and 31.09 × cv. Serr and their parents produced a total of 1.9 × 10^10^ useful reads, ≈2.3 × 10^6^ per hybrid. Low-quality SNPs as defined in Materials and methods and SNPs with more than 40% missing data were removed. Remaining missing SNPs were imputed and wrong allele calls were corrected with the FSFHap algorithm implemented in Tassel v5^22^, which uses parental haplotypes to impute SNPs of progeny. SNPs showing segregation distortion were also removed. A database of SNPs used in linkage map construction including 200 bp of surrounding sequence was created (Supplementary Table 1).

A plot of the first two principal component analysis (PCA) axes revealed the identity of hybrid progenies. Each parent formed a distinct cluster, and hybrids formed two additional clusters (Supplementary Fig. 1). Five and two individuals among the hybrids clustered with *J. microcarpa* and cv. Serr, respectively, rather than with the hybrids, and were excluded since their identity was questionable.

A total of 353 hybrids in the 31.01 × cv. Serr population and 400 hybrids in the 31.09 × cv. Serr population were used in linkage analyses (Table 1, Supplementary Table 2). Segregation of the markers in the two MPs was used to construct linkage maps for the *J. microcarpa* (*Jm)* and *J. regia* (*Jr*) genomes. Each map consisted of 32 linkage groups (LGs), 16 for each parental genome (Supplementary Table 3 and Supplementary Fig. 2).

**Table 1:** Total numbers of GBS SNP markers, the numbers co-segregating marker bins, and the total lengths of linkage maps developed from the *J. microcarpa* 31.01 × cv. Serr and *J. microcarpa* 31.09 × cv. Serr mapping populations

Mapping population	Genome	Markers (no.)	Marker bins (no)	Total length (cM)	Average LG length (cM)	LG length range (cM)
31.01 × cv. Serr	*Jm*	998	882	984.6	61.5	26.1–96.3
	*Jr*	807	672	1326.0	82.9	50.1–125.2
31.09 × cv. Serr	*Jm*	950	619	1115.7	69.7	50.8–101.0
	*Jr*	748	625	1357.4	84.8	53.3–132.3

The linkage map of the *Jm* genome constructed from the 31.01 × cv. Serr MP consisted of 998 SNP markers, which were in 882 co-segregating marker bins, while the linkage map of the *Jr* genome consisted of 807 SNP markers, which were in 672 co-segregating marker bins (Table 1). The total length of the *Jm* genome was 984.6 cM with individual LGs ranging from 26.1 to 96.3 cM and a mean of 61.5 cM (Table 1). The SNP markers adequately covered the linkage groups with a sole exception of LG Jm8S which had a 40 cM gap on the long arm (Supplementary Fig. 2).

The total length of the *Jr* genome, 1326.0 cM (Table 1), was significantly longer than that of the *Jm* genome (*P* = 0.0005, paired *t*-test) with individual LG ranging from 50.1 to 125.2 cM and a mean of 82.9 cM (Table 1 and Supplementary Table 3). The numbers of SNP markers per LG, the lengths of LGs, and the length of corresponding pseudomolecules were positively correlated (Supplementary Table 3).

Similarly, the linkage map of the *Jm* genome constructed from the 31.09 × cv. Serr MP consisted of 950 SNP markers, which were in 619 co-segregating marker bins, whereas that of the *Jr* genome consisted of 748 SNP markers, which were in 625 co-segregating marker bins (Table 1). The total length of the linkage map of the *Jm* genome was 1115.7 cM, but that for the *Jr* genome was longer, 1357.4 cM (Table 1) (*P* = 0.003, paired *t*-test). The individual LGs ranged from 50.8 to 101.0 cM with a mean of 69.7 cM in the *Jm* genome and from 53.3 to 132.3 cM with a mean of 84.8 cM in the *Jr* genome (Table 1, Supplementary Table 3). In contrast to MP 31.01 × cv. Serr, LG Jm8S was adequately covered with SNP markers (Supplementary Fig. 2). The numbers of SNP markers per LG, the genetic lengths of LGs, and the length of corresponding pseudomolecules were positively correlated (Supplementary Table 3).

#### Resistance to *Phytophthora* spp

Resistance to *P. cinnamomi* and *P. pini* was investigated in 15 separate experiments (Supplementary Tables 4, 5). Totals of 249 and 246 hybrids were tested for resistance to *P. cinnamomi* and *P. pini*, respectively, in the 31.01 × cv. Serr population and 223 and 275 were tested for resistance to *P. cinnamomi* and *P. pini*, respectively, in the 31.09 × cv. Serr population (Supplementary Table 4). From 10.2 to 11.5 clones, on average, were screened per hybrid.

Clones grown in soil inoculated with *P. cinnamomi* had on average 33.2 percent of crown length rotted (PCLR) whereas nearly no crown rot was observed on control plants grown in uninoculated soil (Table 2). The difference between inoculated and control plants was less pronounced when percent of root length rotted (PRLR) was employed in the quantification of resistance. Clones grown in soil inoculated with *P. cinnamomi* had on average 44.6 PRLR while plants in control soil had 22.5 PRLR (Table 2).

**Table 2:** Percentages of crown length rotted (PCLR) and percentages of root length rotted (PRLR) of the clones of the F_1_ *J. microcarpa* 31.01 × cv. Serr and *J. microcarpa* 31.09 × cv. Serr hybrids grown in un-inoculated, control soil and clones grown in soil inoculated with *P. cinnamomi* or *P. pini*

Pathogen	Population	Hybrids (no.)	Clones phenotyped per hybrid (no.)	PCLR control soil^a^ (%)	PCLR inoculated soil^a^ (%)	PRLR control soil^a^	PRLR inoculated soil^a^ (%)
*P. cinn*.	31.01 × cv. Serr	249	10.2	1.4a	33.2a	22.5a	44.6a
	31.09 × cv. Serr	223	11.3	2.3a	24.2b	15.5b	34.6b
*P. pini*	31.01 × cv. Serr	246	11.5	1.2a	36.5a	20.5a	36.2a
	31.09 × cv. Serr	275	11.5	2.2a	22.8b	14.2b	27.4b

^a^Values in columns for the same pathogen followed by the same letter do not differ at the *P* < 0.001 (*t*-test) significance level

Clones grown in soil inoculated with *P. pini* had an average of 36.5 PCLR whereas those grown in control soil had an average of 1.2 PCLR (Table 2). However, when PRLR was measured, clones grown in soil inoculated with *P. pini* had an average of 36.2 PRLR and clones grown in control soil had an average of 20.5 PRLR (Table 2). The 31.09 × cv. Serr population of hybrids, on average, had lower PCLR and PRLR means than the 31.01 × cv. Serr population. This was true both for clones grown in control soil (for PRLR variable only) and inoculated soil (for PCLR and PRLR variables) (Table 2).

A Linear Mixed Model (LMM) was built with the PRLR and PCLR variables as described in Materials and Methods. Best Linear Unbiased Predictor (BLUP) values were exported from the fitted model and used in the QTL mapping as phenotypic data.

### Resistance to *A. tumefaciens*

Response to infection with *A. tumefaciens* was analyzed in 429 hybrids and three commercial rootstocks, which were used as controls (Supplementary Table 6). Responses to inoculation were scored on a 1–4 scale, with 1 for resistant and 4 for susceptible. A mean score in population 31.09 × cv. Serr was slightly but significantly lower than a mean score in population 31.01 × cv. Serr (Table 3).

**Table 3:** Mean crown gall score in populations of *J. microcarpa* 31.01 and 31.09 × *J. regia* cv. Serr hybrids

Mapping population	Hybrids (no.)	Clones phenotyped per hybrid (no.)	Mean score^a^
31.09 × cv. Serr	205	5.06	2.18a
31.01 × cv. Serr	224	4.94	2.25b

^a^Shapiro–Wilks and Levene’s tests showed that reaction scores were not normally distributed, and variances were unequal. The non-parametric Mann–Whitney *U* test with Bonferroni correction was used to test the significance of differences between means. Means followed by the same letter are not different at *α* = 0.05

### *P. cinnamomi*, *P. pini*, and *A. tumefaciens* resistance QTL mapping

**Fig. 1:**
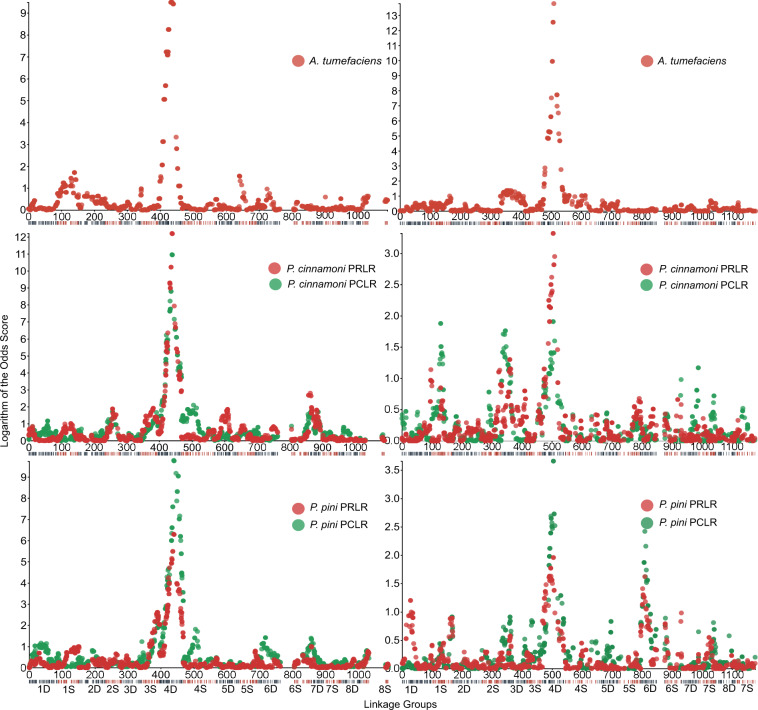
QTL mapping of disease resistance. Scatter diagrams of LOD scores at individual markers across the 16 *J. microcarpa* LGs to infection with *A. tumefaciens*, *P. cinnamoni* and *P. pini* in the 31.01 × cv. Serr (left panels) and 31.09 × cv. Serr (right panels) mapping populations. The QTLs were computed with the IM method

The BLUP PRLR and PCLR values were used in the Interval Mapping (IM) and Multi QTL Mapping (MQM) of *Phytophthora* spp. resistance. Both major and minor QTLs were present on the *J. microcarpa* genetic maps in both mapping populations (Fig. 1); none was present on the two cv. Serr maps. The major QTLs were located on LG Jm4D (see Fig. 1 for IM and Supplementary Fig. 2 for MQM). They were statistically significant in six of the eight QTL analyses of resistance to *Phytophthora* spp. and explained from 12.2 to 22.2% of the variation in a MP. The logarithm of the odds (LOD) of each major QTL peaked in a similar area of LG Jm4D (Table 4 and Fig. 1). None of the minor QTLs was statistically significant (Table 4).

**Table 4:** LOD peaks of QTLs for resistance to *P. cinnamomi, P. pini*, and *A. tumefaciens* mapped with the interval mapping method

Population	Pathogen	Trait	LG	LOD	Significant LOD (cM)	LOD peak location (cM)	% total variation explained
31.01 × cv. Serr	*P. cinn*.	PCLR	Jm4D	10.96^a^	21.3–71.8	43.4	20.2
			Jm4S	2.09	–	36.8	4.2
		PRLR	Jm4D	12.21^a^	22.6–70.3	43.4–43.6	22.2
			Jm7D	2.89	–	3.3	5.8
	*P. pin*.	PCLR	Jm4D	10.24^a^	21.3–71.8	46.7	19
			Jm3S	2.34	–	50.0	4.7
		PRLR	Jm4D	6.31^a^	25.9–63.2	43.2	12.2
			Jm3S	2.61	–	46.7–47.2	5.2
	*A. tum*.	CG	Jm4D	22.1^a^	8.5–57.8	37.43	37.7
31.09 × cv. Serr	*P. cinn*.	PRLR	Jm4D	3.32^a^	22.2–47.3	36.7	7.5
	*P. pin*.	PCLR	Jm4D	3.7^a^	24.4–45.3	36.7	8.3
			Jm6D	2.39	12.4–17.4	13.7	5.5
	*A. tum*.	CG	Jm4D	17^a^	8.8–66	40.5	32.3

^a^Indicate QTLs significant at *α* = 0.05

Responses of commercial rootstocks embedded as standards into each experiment revealed random variation among experiments (Kruskal–Wallis test, *P* < 0.001) (Supplementary Table 5). To determine if this variation affected the QTL analysis, hybrids in each experiment were grouped into two groups based on the allele of a SNP marker closest to the LOD peak of the major QTL. Marker allele *a* was associated with susceptibility to *Phytophthora* spp. and allele *b* with resistance. In most experiments, the two populations of hybrids significantly differed from each other in at least one of the *Phytophthora* sp. × screening method combinations (Supplementary Fig. 3). In an experiment conducted in March 2017 and experiment 2 conducted in July 2017 none of the four *Phytophthora* sp. × screening method combinations yielded a significant difference between the *a* and *b* groups. The two experiments were removed and the QTL analyses, using the IM approach, were repeated (Supplementary Fig. 3). The LOD profiles along the genetic maps of the *Jm* genome remained similar to those when all data were used (compare Fig. 1 with Supplementary Fig. 3), indicating that variation among the experiments did not meaningfully impact the QTL analysis.

A single major QTL was also detected for resistance to *A. tumefaciens* in each MP. The *A. tumefaciens* resistance QTLs were in LG Jm4D, and were in a similar area where the major QTLs for resistance to *Phytophthora* spp. were located. The QTLs were highly significant and explained 32.3 and 37.7% of the variation in *A. tumefaciens* infection response in the two MPs (Table 4). There were two minor peaks of LOD <2, which were inconsistent between the 31.01 × cv. Serr and 31.09 × cv. Serr MPs (Fig. 1) and none was significant.

We propose here the following naming convention for *Juglans* QTLs. A letter *Q* will indicate that a QTL rather than a Mendelian locus is named. *Q* will be followed by a three or four-letter QTL name, followed by the abbreviated name of laboratory that mapped the QTL. The name will end with the name of the chromosome on which the QTL is located. Following this convention, the three major QTLs mapped here were: *QPcin.ucd.Jm4D, QPpin.ucd.Jm4D*, and *QAgr.ucd.Jm4D*. Because the LOD peaks for QTLs based on PCLR and PRLR were co-located, it was assumed that the QTLs mapped with the two resistance screening approaches were estimates of the same QTL.

A null hypothesis that resistance to all three pathogens was associated with the same haplotype was tested by computing correlation coefficients of the resistance scores and the SNP alleles along LG Jm4D (Supplementary Table 7). The correlation coefficients should peak in the same area of the LG and all coefficients should be of the same sign if the hypothesis were true (the alphabetic allele naming used in Supplementary Table 2 was converted into numerical naming, *a* = *1* and *b* = *2* in Supplementary Table 7). On the 31.01 × cv. Serr map, the correlation coefficients peaked between markers *31.01_Jm4D_26359154* and *31.01_Jm4D_26669075* for all three pathogens, and all were negative in that area, indicating that resistance to all three pathogens was associated with haplotype *b*. On the 31.09 × cv. Serr map, the correlation coefficients peaked between markers *31.09_Jm4D_23816262* and *31.09_Jm4D_26359154*, and all correlation coefficients were negative indicating that resistance to all three pathogens was also associated with haplotype *b*.

### Meta-QTL analysis

*QPcin.ucd.Jm4D, QPpin.ucd.Jm4D*, and *QAgr.ucd.Jm4D* were located in the same area of LG Jm4D and resistance to all three pathogens was conferred by the same haplotype, which suggested that resistance to the three pathogens could be conferred by a common Mendelian gene. Since the QTLs were mapped on two different maps built from different SNP markers, the eight individual QTLs could not be directly compared. To facilitate their comparison, a marker closest to each LOD peak was projected onto the Jm4D pseudomolecule^4^ (Table 5). If a LOD peak was between two markers, the arithmetic mean of the marker registry on the Jm4D pseudomolecule was projected. The eight Jm4D projections were used in ANOVA type III analysis to test the null hypothesis that there was no difference in the location of *QPcin.ucd.Jm4D, QPpin.ucd.Jm4D*, and *QAgr.ucd.Jm4D*. The *F*-test was not significant (*P* = 0.239). We, therefore, accepted the null hypothesis that there were no significant differences among the locations of *QPcin.ucd.Jm4D, QPpin.ucd.Jm4D*, and *QAgr.ucd.Jm4D*. The eight projections in bp were averaged (25,502,498 bp) (Table 5) and a 95% confidence interval (CI, Student *t*-distribution) was computed. The CI ranges from 24,547,096 to 26,457,899 bp and contains 158 high-confidence genes, from J*m4DG00123100* to *Jm4DG00138900*^4^.

**Table 5:** Meta-QTL analysis of reactions to infection with *P. cinnamomi, P. pini*, or *A. tumefaciens* in linkage group Jm4D in the *J. microcarpa* × cv. Serr hybrids

QTL	Population	Trait	Markers delimiting LOD peaks^a^	Projection on Jm4D (bp)	Mean projection of all LOD peaks (bp)
*QPcin.ucd.Jm4D*	31.01 × cv. Serr	PCLR	31.01_Jm4D_26168643	26168643	25502498
		PRLR	31.01_Jm4D_26168643	26168643	
	31.09 × cv. Serr	PRLR	31.09_Jm4D_23816262	24037763	
			31.09_Jm4D_24259264		
*QPpin.ucd.Jm4D*	31.01 × cv. Serr	PCLR	31.01_Jm4D_26359154	27006600	
			31.01_Jm4D_27654046		
		PRLR	31.01_Jm4D_25235336	25701990	
			31.01_Jm4D_26168643		
	31.09 × cv. Serr	PCLR	31.09_Jm4D_23816262	24037763	
			31.09_Jm4D_24259264		
*QAgr.ucd.Jm4D*	31.01 × cv. Serr	CG	31.01_Jm4D_23843516	24539426	
			31.01_Jm4D_25235336		
	31.09 × cv. Serr	CG	31.09_Jm4D_26359154	26359154	

^a^If a LOD peak was between markers, the two markers are listed above each other

## Discussion

In our previous studies, progeny of *J. microcarpa* half-sib trees 31.01 and 31.09 segregated for resistance to *Agrobacterum* and *Phytophthora* spp.^2,3^, but the genetic basis was unknown. The two trees were crossed here with *J. regia* cv. Serr and large populations of hybrids for mapping disease resistance were developed. The hybrids were genotyped following the GBS approach^20^ and four genetic maps were produced, one each for the *Jm* 31.01 and *Jm* 31.09 genome and two for *J. regia* cv. Serr. The maps were deployed in QTL mapping of resistance to *A. tumefaciens*, *P. cinnamomi*, and *P. pini*.

### Genetic maps

About 20% more SNPs were obtained in the *Jm* genome than in the *Jr* genome in both MPs, which was consistent with greater nucleotide diversity observed in *J. microcarpa* (*π* = 8.4 × 10^–3^) compared to that in *J. regia* (*π* = 5.6 × 10^−3^)^19^. Maps of the *Jm* genome were consequently built from greater numbers of markers than the cv. Serr maps. Even low genotyping and mapping error rates lead to substantial map length expansions^23^, which increase as the numbers of markers on such maps increase. *J. microcarpa* maps would be longer than the cv. Serr maps if our deployment of the GBS were burdened by errors since the *J. microcarpa* maps were built from larger numbers of markers than the cv. Serr maps. Both *J. microcarpa* maps were shorter than the cv. Serr maps, providing no evidence of map expansion, indicating that we were successful in keeping GBS error rates low.

The lengths of the cv. Serr maps, in addition to being longer than the *J. microcarpa* maps, also exceeded the lengths of the maps of *J. regia* cv. Chandler published earlier^5,20,24^. Chandler, like the *J. microcarpa* mother trees 31.01 and 31.09, was used as the female parent. Sex-related differences in recombination rates are common in both plants and animals. Our data suggest that recombination rates are greater in the male meiosis than in the female meiosis in *Juglans*.

The short lengths of LGs is another notable feature of recombination in *Juglans* genomes^5^. On average, the genetic lengths of the *Juglans* chromosomes are about half of the genetic lengths typically found for chromosomes in herbs, particularly those that are self-pollinating^5^. Within-arm double crossovers (DCO) take place rarely in *Juglans*. Analyses of our four maps estimated intra-arm DCO rates to range from 4.15 to 7.26% meiotic chromosome arms (Supplementary Table 8). *Juglans* meiosis is thus characterized by distal crossover localization^5,25^ and paucity of DCO. These two tendencies produce genetically short chromosomes with distally located crossovers.

### Resistance QTLs

Both *J. microcarpa* mother trees were heterozygous for QTLs on chromosome Jm4D conferring resistance to each of the three investigated pathogens. QTL *QAgr.ucd.Jm4D* explained 32.3 and 37.7% of phenotypic variation in the 31.01 × cv. Serr and 31.09 × cv. Serr populations, respectively. In contrast, QTLs *QPcin.ucd.Jm4D* and *QPpin.ucd.Jm4D* explained only from 8.2 to 19.3% of phenotypic variation. The lower percentage of phenotypic variation explained by the *Phytophthora* spp. QTLs were likely related to random variation that accompanied the PCLR and PRLR assays. Another source of variation could have been individual screening “experiments”. Nearly 20,000 clones were screened for resistance to *Phytophthora* spp., which necessitated screening for resistance to *Phytophthora* spp. over a period of four years in 15 different times (experiments). To reduce random variation due to this source, clones of three rootstocks were embedded as standards in each experiment, a mixed linear model was built, and BLUP values were computed and used in QTL mapping as phenotypic data. Our *posteriori* analysis of the QTL data revealed that this approach minimized the experiment effect on the results of the QTL analysis.

The LOD score for the *QAgr.ucd.Jm4D, QPcin.ucd.Jm4D*, and *QPpin.ucd.Jm4D* QTLs peaked in the same general area of LG Jm4D and resistance scores for all three pathogens correlated with haplotype *b* in both MPs. We, therefore, hypothesized that the resistance to all three pathogens had a common genetic cause. Meta-QTL analysis placed with 95% probability this hypothetical locus into an interval 24,547,096–26,457,899 bp on pseudomolecule Jm4D. This interval contains 158 high-confidence genes, from *Jm4DG00123100* to *Jm4DG00138900*.

It could be that the co-location of the three QTLs is just a coincidence caused by high density of resistance genes in that region of the Jm4D chromosome. This region of the *Jm* genome has been shown to harbor one of the highest concentrations of resistance genes and some are in the Jm4DG00123100 to Jm4DG00138900 interval^4^. Alternatively, the co-location of resistance to these different pathogens could be due to broad spectrum resistance (BSR) conferred by a single locus^26^. There are numerous examples in which resistance to multiple diseases has appeared to be co-located on a map, but unequivocal evidence for BSR requires isolation of the resistance gene, which we have not done, and cannot decide between these two alternatives.

In an attempt to find a candidate gene for *Phytophthora* spp. resistance, the QTL for resistance to *P. cinnamomi* on Jm4D was compared to QTLs for resistance to *P. cinnamomi* mapped in Japanese and Chinese chestnuts. In both chestnut species, resistance to *P. cinnamomi* was mapped in LGs E and K^15,16^ indicating conservation of the two QTLs in *Castanea*. We hypothesized that if the region on Jm4D harboring the major QTL is homoeologous to any of the two *Castanea* regions, shared genes could suggest candidate resistance genes. Homology was examined for 247 genes located in the region from 24 to 27 Mb on the Jm4D pseudomolecule against scaffold sequences of Chinese chestnut https://www.hardwoodgenomics.org/Genome-assembly/1962958 at the QTLs on LGs E and K. Only one of the 247 genes, a resistance analogue *Jm4DG00140500*, was shared indicating *J. microcarpa* and *Castanea* QTLs were not orthologous.

### Rootstock breeding

Crown gall and Phytophthora root and crown rots are devastating soil-borne diseases of commercial walnut orchards in California. Currently, California rootstocks have some resistance to single pathogens^2,3^, but none is known to be highly resistant to two or more pathogens. A remarkable feature of resistance segregating in the progeny of *J. microcarpa* trees 31.01 and 31.09 is that resistance to *A. tumefaciens* and both *Phytophthora* spp. is mediated by the same region of chromosome Jm4D and by the same haplotype. These attributes and availability of SNP markers for selection of this resistance (Supplementary Table 1) will greatly simplify stacking resistance to both *A. tumefaciens* and *Phytophthora* spp. in a single rootstock.

## Materials and methods

### Plant materials

*Juglans microcarpa* trees DJUG 31.01 and DJUG 31.09 (USDA National Clonal Germplasm Repository, Davis, https://www.ars.usda.gov/pacific-west-area/davis-ca/natl-clonal-germplasm-rep-tree-fruit-nut-crops-grapes/) were used as mother trees in hybridization with *J. regia* cv. Serr in the springs of 2012–2015. Female flowers were sealed in pollen-impermeable semi-porous bags (PBS 10-1, PBS International) prior to opening, using non-absorbent cotton to cushion and seal the bags around the branch after male flowers had been removed. When female flowers began opening (at the stigma separation stage), previously collected cv. Serr pollen was injected into the bags using a syringe. The bags were removed 3–4 weeks after pollination and immature nuts were tagged. The nuts were collected while still immature and with intact hulls (July–August), and stored refrigerated (up to several weeks). Embryos were extracted from nuts, germinated in vitro, and micropropagated^27^ to produce clones for disease resistance testing.

### Resistance to *P. cinnamomi* and *P. pini*

**Fig. 2:**
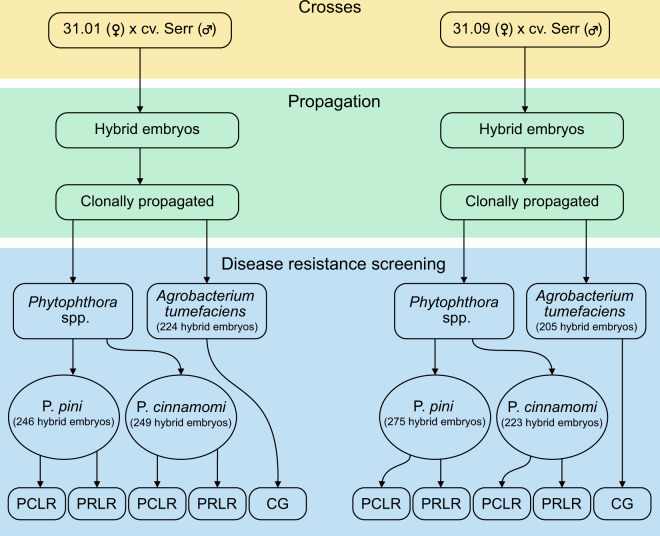
Production, clonal propagation, and disease screening of *J. microcarpa* × *J. regia* hybrids. The bottom row indicates trait abbreviations: percent crown length rotted (PCLR), percent root length rotted (PRLR), and crown gall resistance score (CG)

Rooted clonal plants derived from 31.01 × cv. Serr and 31.09 × cv. Serr hybrids were transferred to 0.5-L pots containing UC potting mix^28^ and grown in a greenhouse. When the plants reached at least 15 cm in height, they were entered into greenhouse evaluations of resistance to *Phytophthora* spp. The numbers of hybrid progeny evaluated for resistance to *P. cinnamomi* and *P. pini* (formerly *P. citricola sensu lato*) across 15 experiments are specified in Fig. 2. Each experiment included three clonal rootstocks with known levels of resistance as standards: ‘AX1’ (low resistance to both *P. cinnamomi* and *P. pini*), *J. microcarpa* × *J. regia* ‘RX1’ (highly resistant to *P. cinnamomi* and moderately resistant to *P. pini*), and wingnut (*Pterocarya* sp) ‘WNxW’ (highly resistant to both *P. cinnamomi* and *P. pini*). Evaluations of resistance to *Phytophthora* spp. were avoided during fall and winter (November–January), because it was determined that susceptibility to *Phytophthora* spp. tends to be reduced during that period.

Four isolates of *P. cinnamomi* (GB647, GB5972, GB5973, and GB6378) were combined for inoculation. Likewise, four isolates of *P. pini* (GB572, GB5181, GB5781a, and GB5997k) were combined for inoculation. Plants were inoculated either with *P. cinnamomi* or *P. pini*. All of the isolates were from walnut trees affected by Phytophthora root and crown rot in the Central Valley of California and had been hyphal tipped. Identity of the isolates was confirmed by sequencing the rRNA gene internal transcribed spacer (ITS)^2^. All of the isolates possessed morphological traits (e.g., sporangia shape and papilla type, hyphal morphology, and the presence and morphology of oogonia and antheridia) characteristic of their identity. The combination of four isolates was used for each *Phytophthora* sp. to ensure that resistance determined in the greenhouse would also be expressed in California orchards. There is no evidence for the existence of *Phytophthora* races in California walnut orchards or walnut cultivar × *Phytophthora* isolate interactions for either *P. cinnamomi* or *P. pini*. The use of multiple Phytophthora isolates to represent a *Phytophthora* sp. is a common practice in *Phytophthora* sp. resistance QTL studies in woody perennials, e.g.^16^. Similarly, QTLs for resistance to *P. cactorum* in strawberry were mapped using multiple-isolate mixtures^29^.

Inoculum was prepared, plants were inoculated, and an experimental design previously described^2^ for phase 2 screening was used. Two months after inoculation, PCLR and PRLR were assessed as described previously^2^. Also, after the disease assessments, culture-based isolations were used as described previously^2^ to determine that plants grown in non-infested soil had remained free from Phytophthora and that those grown in soil infested with *P. cinnamomi* or *P. pini* had been exposed to the appropriate pathogen.

In statistical analysis, PCLR and PRLR values from *Phytophthora* sp.-inoculated plants were compared to those from control plants using the Mann–Whitney *U* test. Resistance among hybrids was evaluated across multiple greenhouse experiments. A Kruskal–Wallis test was used to assess the effect of experiment on phenotypes. Linear Mixed Model (LMM) implemented in lme4 v1.1.18.1 R package^30^ was used to assess the effects of random variables on the response variable. Genotype and experiment were random variables in the LMM model, while the disease phenotype scores were response variables. Normality of fitted model residuals was visualized using a histogram and tested using Shapiro–Wilk test. BLUP values were extracted from the model and used in-place of phenotype values in the QTL analysis.

### Resistance to *A. tumefaciens*

Rooted clonal plants derived from 31.01 × cv. Serr and 31.09 × cv. Serr hybrids were transferred to 0.5-L pots containing UC potting mix and cultivated in the greenhouse until the stems were at least 0.5 cm in diameter. Three to six clonal plants per hybrid were used for *A. tumefaciens* resistance screening. Commercial walnut rootstocks VX211, Vlach (*J. hindsii* × *J. regia*), and RX1 were used as standards. Groups of nine unique clones, i.e., each derived from a different hybrid or check, were randomly selected and placed into plastic bins. Bins were randomized on a greenhouse bench.

Colonies of *A. tumefaciens* strain EC1 were grown for 48 h at 28 °C on tryptic soy agar (TSA). Ten mL of tryptic soy broth (TSB) was inoculated with a single colony and grown overnight on a shaker at 210 rpm and 28 °C. The TSB culture was pelleted in a Sorvall RC 5C Plus centrifuge (Fisher Scientific, Waltham, MA) at 4575 g in a SH3000 swinging bucket rotor at room temperature. The bacterial cell pellet was washed twice with sterile water. The pellet was suspended in sterile water and adjusted to absorbance 600_nm_ = 1.0 (~10^9^ CFU/ml) using a Beckman DU 800 spectrophotometer (Beckman, Fullerton, CA).

Two joined 0.9 cm metal Exacto blades (ACE hardware, Oak Brook, IL) were dipped into 2 mL of an *A. tumefaciens* suspension, and the *A. tumefaciens* infested blades were pierced 2 mm deep into the main stem at two positions approximately 0.5 cm above the soil line. The blade was dipped again into the suspension and a second stab wound was made on the stem, 2.5 cm above the first stab wound. The resulting vertical stabs were wrapped with parafilm. A single clone per hybrid was stabbed with a blade dipped in sterile water, which served as a negative control. Susceptible hosts, *Datura stramonium* and *Solanum lycopersicum* (tomato), were inoculated with the same *A. tumefaciens* EC1 suspension, which served as positive controls. Plants were grown in the greenhouse with natural light supplemented with 16 h of overhead LED lighting daily and temperature 10–22 °C during the day and 10–17 °C at night.

Plants were examined for gall development two months after inoculation. The following numerical system was used to record the percentage of stem girdling by gall tissue: 1 = no gall or tumor, 2 = <25% of stem girdled, 3 = 25–50% stem girdled, and 4 = >50% stem girdled. A rating of 1 indicated a highly resistant/tolerant response. A rating of 2 indicated a partially resistant response, and 3 and 4 indicated susceptible and highly susceptible response, respectively.

### GBS and SNP discovery

Genomic DNA was isolated with the CTAB plant genomic DNA isolation method^31^ from micro-propagated F_1_ hybrids and *J. microcarpa* and cv. Serr parents. DNA was diluted to the uniform concentration of 55 ng/µl. GBS involved complexity reduction by *PstI* restriction enzyme digestion^21^. Groups of 96 samples including both parents were multiplexed using unique barcodes, and single-end sequenced with the Illumina HiSeq2000 platform at the Genomic Diversity Facility of Institute of Biotechnology, Cornell University. SNPs were called with Tassel-GBS pipeline v2.0^32^ using default settings except that a minimum mapping quality of 2 was used in SAMToGBSdbPlugin. Illumina reads were deconvoluted using the barcodes, reads were filtered and trimmed to 64 bp to construct unique tags. The *J. microcarpa* Jm31.01_v1.0 and *J. regia* JrSerr_v1.0 genome sequences^4^ were combined into a single fasta file and used as a reference. Unique tags were aligned onto the reference sequences with the BWA-aln aligner. High-quality SNPs were selected among SNPs obtained from the Tassel-GBS pipeline with VCFtools^33^, BCFtools^34^, and in-house awk and Python scripts. Non-segregating SNPs and calls below a threshold of five reads were removed.

The following convention was implemented in naming the markers. Each name was started with that of the mapping population followed by the abbreviated names of the genome and chromosome, and ended with the location of the SNP in the registry of the corresponding JrSerr_v1.0 or Jm31.01_v1.0 pseudomolecule^4^.

To verify that pollen contamination or some other perturbation had not occurred during the development of hybrids, the SNP data were analyzed with a Principal Component Analysis (PCA) implemented in the Tassel v5.0^22^.

### Linkage map construction

Linkage maps were constructed separately for the *J. microcarpa* and cv. Serr genomes in each mapping population using the Joinmap v4.1 mapping program^35^. SNP markers showing segregation distortion (*P* < 0.001, χ^2^ test) were removed. Remaining markers were grouped into linkage groups (LGs) by a regression mapping procedure^36^ with a minimum threshold logarithm of odds (LOD) score of 8 and a maximum recombination fraction of 0.3. The Joinmap v4.1^35^ assigned sequential numbers to the loci after computing the groupings of co-segregating markers. For a bin of co-segregating markers, the first marker in a bin was chosen as the representative marker of the bin by the mapping program. Recombination fractions were converted into map distances (cM) with the Kosambi mapping function^37^. Marker order along each LG was confirmed by comparing it with the order along the reference genome pseudomolecule.

### QTL analysis

QTLs were mapped separately in each mapping population with MapQTL v6.0^38^. IM with one cM increments was employed to locate putative QTLs. QTLs with significant LOD threshold (*α* = 0.05) values were identified with a permutation test with 1000 independent permutations. SNPs exceeding significant threshold LOD (*α* = 0.05) were used as initial cofactors (https://www.kyazma.nl/docs/MQ6Manual.pdf). Cofactors that were significant were identified with the automatic cofactor identification method and used in MQM^39,40^. Percentage of variance explained by each QTL and their additive genetic effects were extracted from MQM.

### Statistical analysis and data visualization

Statistical tests were performed using either R^41^ or Python SciPy v1.0 library^42^. Linkage maps were drawn using our in-house JavaScript library built on top of d3js^43^. Bar graphs and histograms were drawn using R, Matplotlib^44^, or Seaborn Python (http://seaborn.pydata.org) packages.

## Supplementary information

Supplementary Table 1

Supplementary Table 2

Supplementary Table 3

Supplementary Table 4

Supplementary Table 5

Supplementary Table 6

Supplementary Table 7

Supplementary Table 8

Supplementary Figure 1

Supplementary Figure 2

Supplementary Figure 3

## Data Availability

All data generated in this study are included in this published article and its supplementary information.
