# Supplementary material for: Co-located quantitative trait loci mediate resistance to Agrobacterium tumefaciens, Phytophthora cinnamomi, and P. pini in Juglans microcarpa × J. regia hybrids
Source: Hortic Res. 2021 May 1;8:111. doi: 10.1038/s41438-021-00546-7 (PMC8087670; doi:10.1038/s41438-021-00546-7)
Supplement: Supplementary file 5 — Supplementary Table 5 [file 41438_2021_546_MOESM5_ESM.docx]

**Supplementary Table 5**

Resistance to *Phytophthora* cinnamomi and *P. pini* was investigated in15 separate experiments conducted over a three-year period, June, 2014 to September 2017 (**Table 1**). Rootstocks RX1 and AX1 were used as checks in each of the 15 experiments. Descriptive statistics of PCLR and PRLR values show great environmental variation in scores both within and between the 15 experiments (**Figs 1 to 4**).

Table S1. Numbers of checks included in each of the 15 separate evaluation of resistance of 31.01 x Serr and 31.09 x Serr hybrids to *Phytophthora cinnamomi* and *P. pini*

| Experiment (date) | AX1 rootstock (no.) | RX1 rootstock (no.) |
| --- | --- | --- |
| 2014Janex1 | 10 | 10 |
| 2014Julex3 | 15 | 15 |
| 2014Junex2 | 15 | 15 |
| 2015Jun | 15 | 15 |
| 2016Aug | 10 | 10 |
| 2016Jun | 12 | 12 |
| 2016May | 15 | 15 |
| 2017Apr1 | 15 | 15 |
| 2017Apr2 | 15 | 15 |
| 2017July1 | 15 | 15 |
| 2017July2 | 15 | 15 |
| 2017June1 | 15 | 15 |
| 2017June2 | 15 | 15 |
| 2017Mar | 15 | 15 |
| 2017Sep | 15 | 15 |


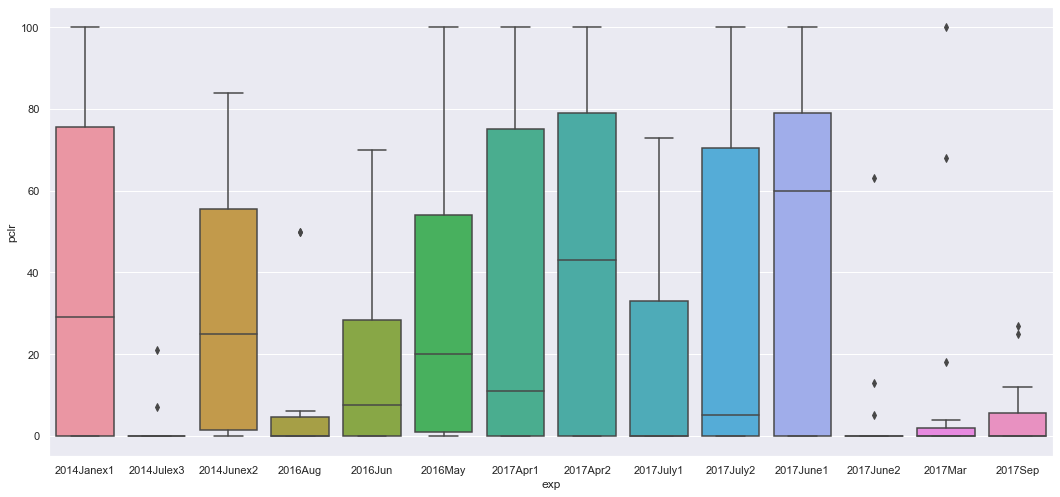


**Fig. S1.** Boxplots showing median PCLR score, Q1 and Q3, and minimum and maximum PCLR score in commercial rootstock RX1 used as checks across the 15 experiments.


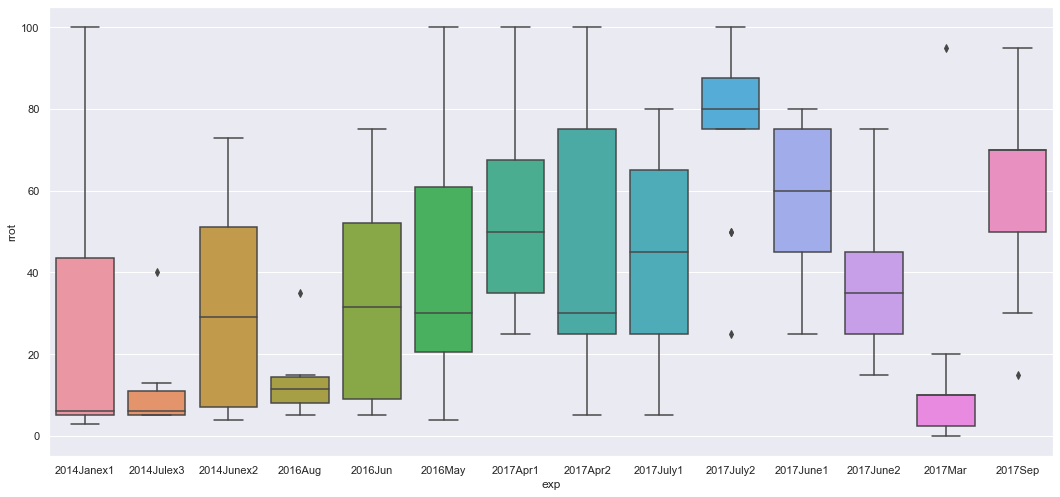


**Fig. S2.** Boxplots showing median PRLR score, Q1 and Q3, and minimum and maximum PRLR scores in commercial rootstock RX1 used as checks across the 15 experiments.


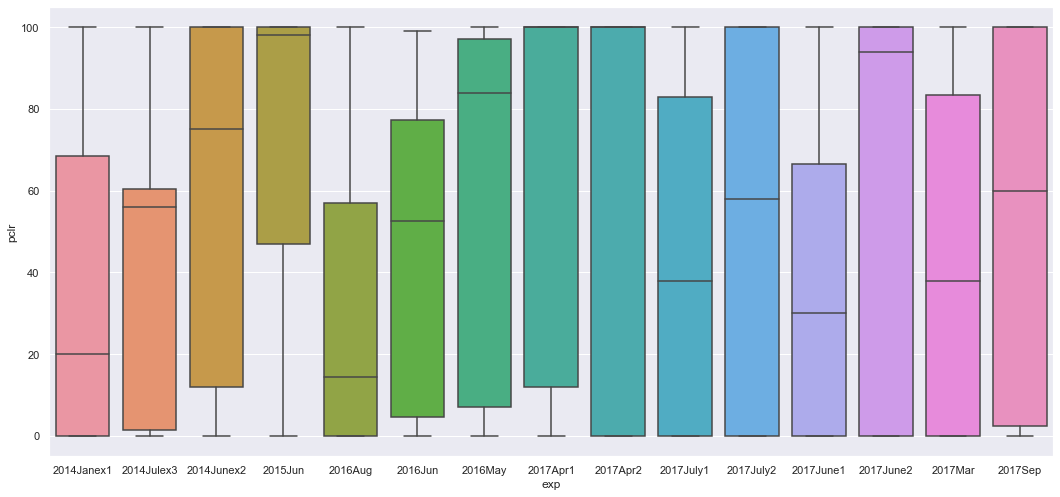


**Fig. S3.** Boxplots showing median PCLR score, Q1 and Q3, and minimum and maximum PCLR scores in commercial rootstock AX1 used as checks across the 15 experiments.


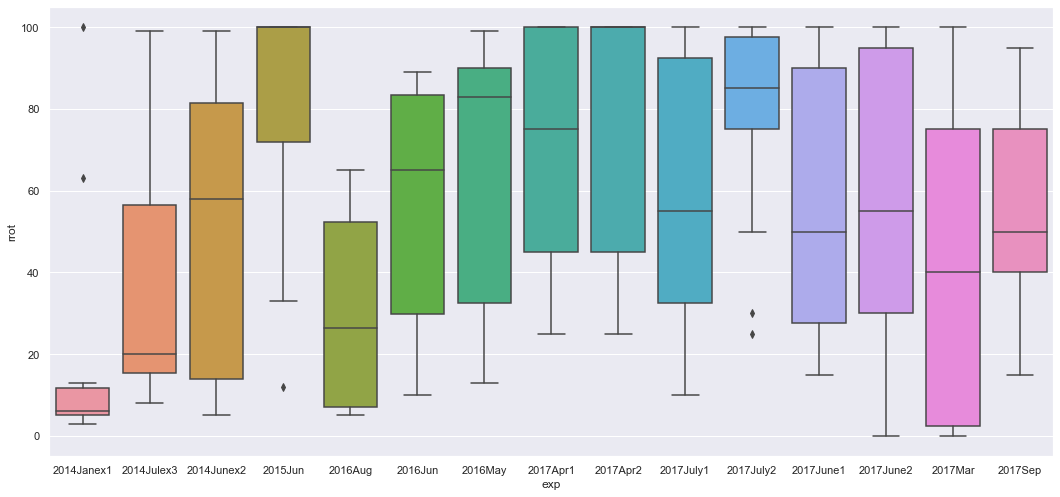


**Fig. S4**. Boxplots showing median PCLR score, Q1 and Q3, and minimum and maximum PCLR scores in commercial rootstock AX1 used as checks across the 15 experiments.
