# Supplementary material for: Co-located quantitative trait loci mediate resistance to Agrobacterium tumefaciens, Phytophthora cinnamomi, and P. pini in Juglans microcarpa × J. regia hybrids
Source: Hortic Res. 2021 May 1;8:111. doi: 10.1038/s41438-021-00546-7 (PMC8087670; doi:10.1038/s41438-021-00546-7)
Supplement: Supplementary file 8 — Supplementary Table 8 [file 41438_2021_546_MOESM8_ESM.docx]

**Supplementary Table 8**. Probability for double crossover (DCO) taking place within a meiotic chromosome arms in *J. microcarpa* 31.01 and 31.09 and *J. regia* cv Serr

GBS data were available for 11296 and 13120 chromosome arms per genome in the 31.01 × cv Serr and 31.09 × cv Serr mapping populations (**Supplementary Table 2**). The centromeres in the *J. microcarpa* and *J. regia* pseudomolecules^4^ was used to delimit chromosome arms in LGs in the **Supplementary Table 2** data. Chromosome arms that showed a DCO involving fewer than 6 SNPs were disregarded, since they could have been genotyping or imputation errors. In the remaining chromosome arms a DCO was present in 136 to 205 arms per genome (**Table S1**). There are four ways a double crossover can take place in a meiotic bivalent. Of the 16 chromatics (4 chromatids x 4 bivalents) one-quarter chromatids show a DCO. Thus, the observed frequency of DCO in progeny chromosomes should be multiplied by 4 to estimate the actual percent of DCO per meiotic chromosome arm (**Table S1**).

Table S1.

| Mapping population | Genome | Chr. arms investigated (No.) | Chromosome with DCO (No.) | Observed chrom. arms with DCO (%) | Estimated DCO per meiotic chrom. arm  (%) |
| --- | --- | --- | --- | --- | --- |
| 31.01 x cv Serr | *Jm* | 11296 | 205 | 1.81 | 7.26 |
|  | *Jr* | 11296 | 178 | 1.58 | 6.30 |
| 31.09 x cv Serr | *Jm* | 13120 | 152 | 1.16 | 4.63 |
|  | *Jr* | 13120 | 136 | 1.04 | 4.15 |
