# Supplementary material for: Co-located quantitative trait loci mediate resistance to Agrobacterium tumefaciens, Phytophthora cinnamomi, and P. pini in Juglans microcarpa × J. regia hybrids
Source: Hortic Res. 2021 May 1;8:111. doi: 10.1038/s41438-021-00546-7 (PMC8087670; doi:10.1038/s41438-021-00546-7)
Supplement: Supplementary file 9 — Supplementary Figure 1 [file 41438_2021_546_MOESM9_ESM.docx]

**Supplementary Fig. 1.** Principal component analysis of *J. microcarpa* 31.01 *× J. regia* cv Serr and *J. microcarpa* 31.09 × *J. regia* cv Serr mapping populations


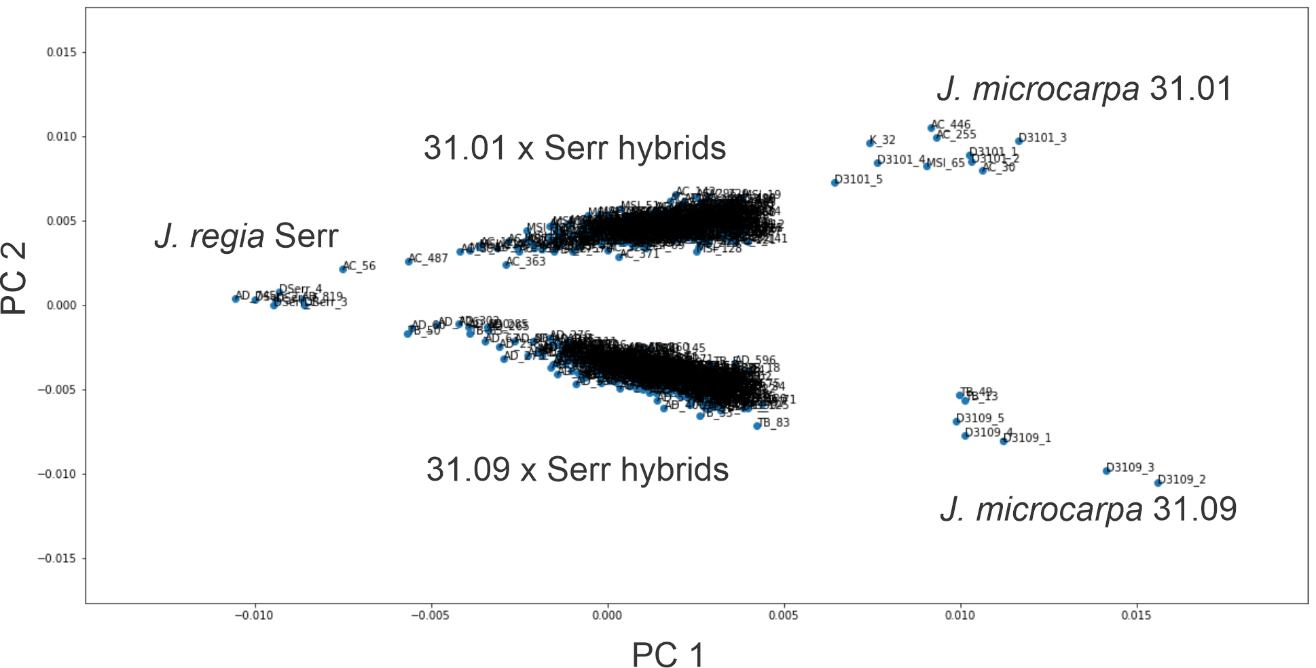


PCA using the genotypes of *J. regia* cv Serr and *J. microcarpa* mother trees 31.01 and 31.09 and putative hybrids 31.01 x Serr and 31.09 x Serr, respectively. Putative hybrids AC_30, AC_446, AC_255, TB_13, and TB_49 clustered with *J. microcarpa* rather than with a hybrid cluster, which indicated that they were self-pollinated progeny. Putative hybrids AD_745 and AD_819 clustered with Serr, which indicated that they were not true hybrids.
