# Supplementary material for: Co-located quantitative trait loci mediate resistance to Agrobacterium tumefaciens, Phytophthora cinnamomi, and P. pini in Juglans microcarpa × J. regia hybrids
Source: Hortic Res. 2021 May 1;8:111. doi: 10.1038/s41438-021-00546-7 (PMC8087670; doi:10.1038/s41438-021-00546-7)

**Supplementary Fig. 2.** Locations of *A. tumefaciens* and *Phytophthora* resistance QTLs on genetic maps of *J. microcarpa* and *J. regia* constructed from hybrid mapping populations 31.01 × cv. Serr and 31.09 × cv. Serr. The rulers indicate the genetic lengths of linkage groups (LGs), horizontal bars indicate the locations of SNP marker in LGs, and colored vertical bars indicate the position of disease resistance QTLs located by the MQM method. The vertical bars span markers at which LOD is above a threshold LOD value.


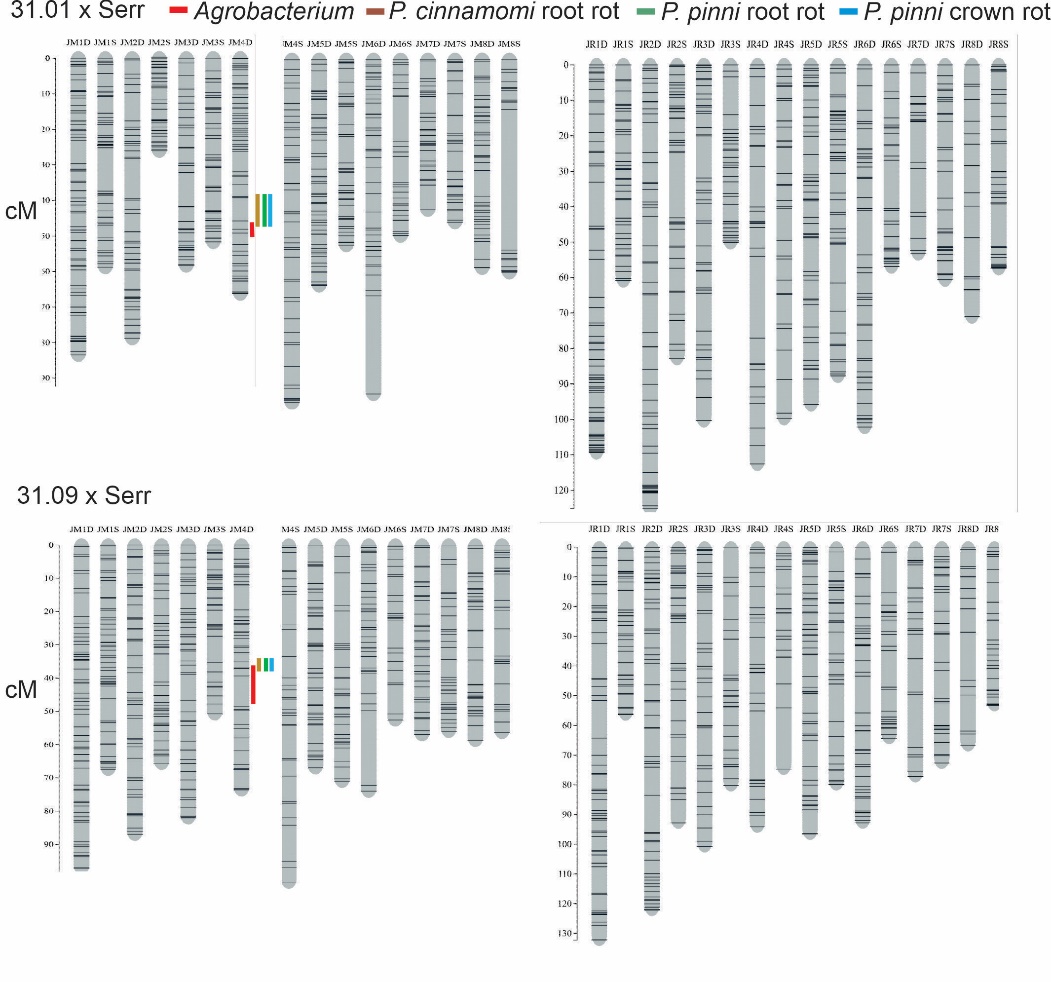

Supplement: Supplementary file 10 — Supplementary Figure 2 [file 41438_2021_546_MOESM10_ESM.docx]
