# Supplementary material for: Co-located quantitative trait loci mediate resistance to Agrobacterium tumefaciens, Phytophthora cinnamomi, and P. pini in Juglans microcarpa × J. regia hybrids
Source: Hortic Res. 2021 May 1;8:111. doi: 10.1038/s41438-021-00546-7 (PMC8087670; doi:10.1038/s41438-021-00546-7)
Supplement: Supplementary file 11 — Supplementary Figure 3 [file 41438_2021_546_MOESM11_ESM.docx]

**Supplementary Fig. 3. Variation among individual *Phytophthora* experiments and QTL analysis with a subset of data**

Clones of hybrids in each of the 15 screening for *Phytophthora* resistance among the hybrids (experiments) were allocated into the *a* and *b* haplotype groups based on the allele of markers below the LOD peaks in the QTL analysis described in the paper. Percent of crown length rotted (PCLR) and percent of root length rotted (PRLR) were used as variables in one-way ANOVA in each experiment to compute the *P*-value of the difference in reaction to infection between the *a* (susceptible) and *b* (resistant) haplotype groups (**Table S1 below**). In two experiments (2017Mar and 2017July2) the difference was not significant in any of the four combinations of *Phytophthora* spp. × screening assay (PCLR or PRLR). The two sets of data were excluded since it is likely that conditions of the experiment precluded expression of resistance, and QTLs were recomputed using the interval methods from the remaining data (**Fig. S1 below**). While the heights of Jm4D LOD peak values increased the LOD profile remained similar to that in **Fig. 1** in the paper.

**Table S3**. *P*-values (Kruskal test) of differences in reaction to infection with *P. pini* and *P. cinnamomi* between hybrids with haplotype *a* and haplotype *b* in the 15 separate experiments

|  | Experiment | Hybrids (No.) | *P. pini* | *P. pini* | *P. cinnamomi* | *P. cinnamomi* |
| --- | --- | --- | --- | --- | --- | --- |
|  |  |  | PCLR | PRLR | PCLR | PRLR |
| 31.01 × Serr | 2017July1 | 13 | 0.3967 | 0.0200 | 0.1302 | 0.0142 |
|  | 2017June1 | 19 | 0.0000 | 0.0000 | 0.0000 | 0.0000 |
|  | 2017Sep | 55 | 0.0000 | 0.0000 | 0.9435 | 0.0002 |
|  | 2017July2 | 17 | 0.5131 | 0.0055 | 0.6178 | 0.0003 |
|  | 2017Apr1 | 37 | 0.0000 | 0.0000 | 0.0000 | 0.0000 |
|  | 2017Mar | 23 | 0.4616 | 0.7377 | 0.3976 | 0.9720 |
|  | 2017Apr2 | 11 | 0.0003 | 0.0331 | 0.0001 | 0.0000 |
|  | 2017June2 | 7 | 0.1473 | 0.0001 | 0.0255 | 0.0001 |
|  | 2014Junex2 | 43 | 0.0000 | 0.0098 | 0.0000 | 0.0445 |
|  | 2014Janex1 | 31 | 0.0134 | 0.0109 |  |  |
| 31.09 × Serr | 2017Mar | 47 | 0.0000 | 0.0000 | 0.0763 | 0.0001 |
|  | 2014Julex3 | 35 | 0.0000 | 0.9023 | 0.0000 | 0.0000 |
|  | 2017Apr2 | 19 | 0.0000 | 0.0021 | 0.0000 | 0.0000 |
|  | 2017June2 | 16 | 0.1874 | 0.0739 | 0.4074 | 0.0137 |
|  | 2017Sep | 14 | 0.0187 | 0.0230 | 0.1753 | 0.1885 |
|  | 2017July2 | 6 | 0.7761 | 0.6617 | 0.3276 | 1.0000 |
|  | 2017June1 | 15 | 0.0003 | 0.0000 | 0.0000 | 0.0000 |
|  | 2016Aug | 62 | 0.0000 | 0.0000 |  |  |
|  | 2016Jun | 16 | 0.0259 | 0.2702 | 0.3436 | 0.0247 |
|  | 2016May | 12 | 0.1643 | 0.1267 | 0.3769 | 0.0647 |


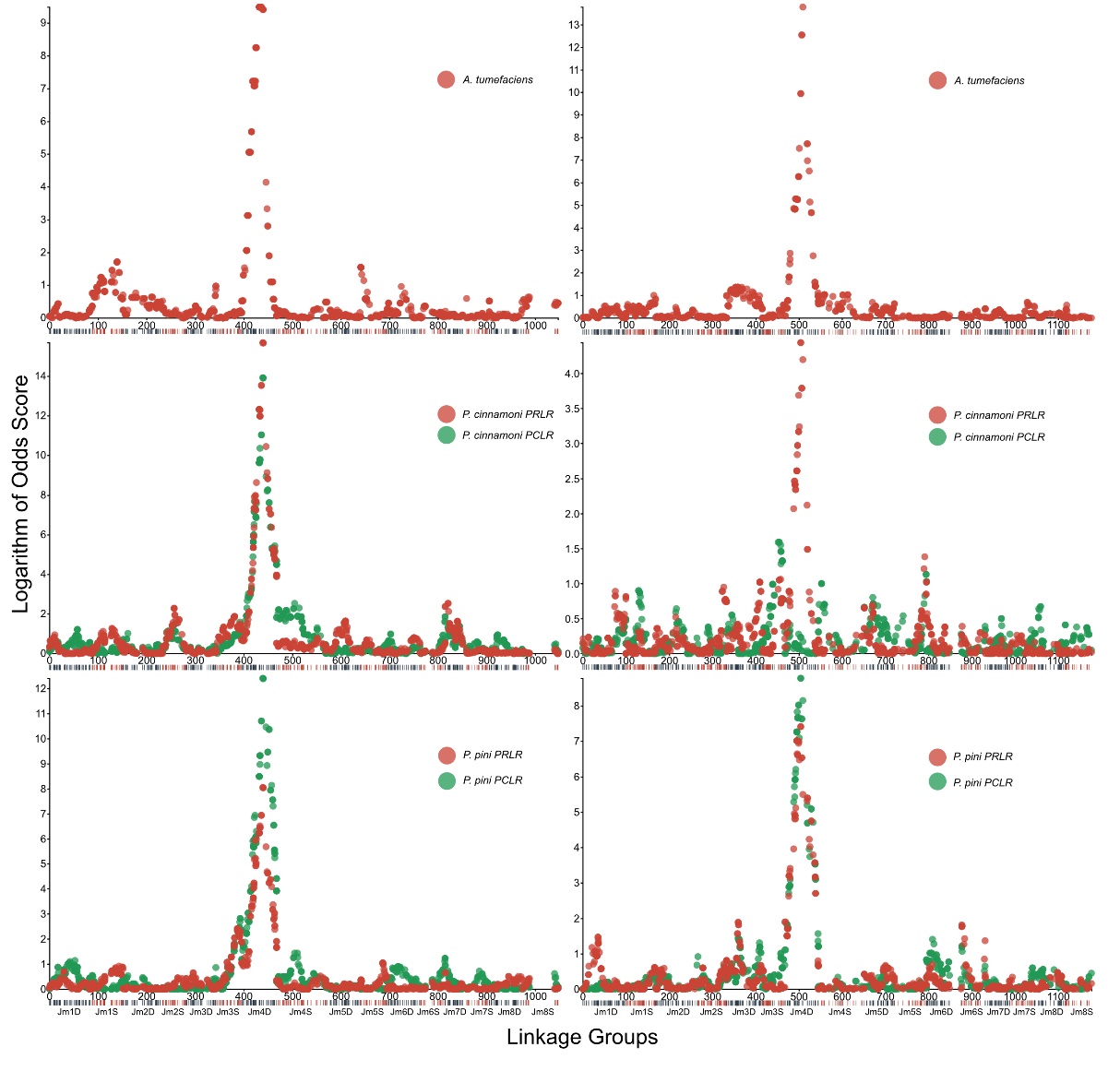


**Fig. S3**. Scatter diagrams of LOD scores at individual markers on linkage maps of reaction to infection by *A. tumefaciens*, *P. cinnamoni* and *P. pini* across the *J. microcarpa* linkage map in the 31.01 × cv. Serr (left panels) and 31.09 × cv. Serr (right panels) populations. The QTLs were computed with the interval method. Cumulative linkage map in cM, LG name, and LG genetic map for the D subgenomes (black) and S subgenomes (red) are indicated below the *x*-axis.
